# Fabrication and Use of a Customized Provisional Composite Abutment in Dental Practice

**DOI:** 10.1155/2021/9929803

**Published:** 2021-08-21

**Authors:** Roman Studenikin, Sabukhi Niftaliev

**Affiliations:** ^1^Dental Clinic Vash Stomatolog, Bulvar Pionerov,17B, Voronezh 394038, Russia; ^2^Voronezh State University of Engineering Technologies, Pr. Revolutsii,19, Voronezh 394036, Russia

## Abstract

**Introduction:**

Reducing the time of implant integration and the period of prosthetics is an important task of dentistry since this leads to improved quality of life and successful rehabilitation of the patient. Therefore, currently, there is an intensely increased interest in immediate or early loading of the implant, when certain parameters of primary implant stability in the bone tissue are achieved.

**Materials and Methods:**

The materials used to perform the procedure for placement of a customized provisional composite abutment were a provisional prefabricated abutment with a retention grip for the composite; aluminum oxide powder with a particle size of 27 *μ*m for better adhesion of the composite, with which the retention grip of the provisional abutment is coated; 3M Single Bond Universal light-curing adhesive applied to the provisional abutment; and Filtek Bulk Fill 3M composite including a low-viscosity radiopaque nanocomponent and ytterbium trifluoride filler with a particle size of 0.01–3.5 nm. Methods used in this study were as follows: fabrication technique using the Cervico system for a customized provisional composite abutment; sandblasting of the provisional abutment using the apparatus RONDOFLEX (KERR); light polymerization of low-viscosity composite using Demi Ultra Kerr lamp (luminous flux power not less than 1100 mW/cm^2^); and radiographic control of the abutment fit in the implant.

**Results:**

The surgical and orthopedic treatment of 20 patients was performed using this technique. The control group consisted of 11 patients with similar pathology, in whose surgery the fabrication of a provisional prosthesis was used. As a result, it was possible to form a gingival profile, in comparison with the control group, to accelerate mucogingival and bone integration, as well as to quickly carry out orthopedic rehabilitation of the patient. The average value of the time required for the final formation of soft tissues for prosthetics in patients in the experimental group was significantly lower than those in the comparison group (*p*=0.007 and *p*=0.028, respectively). In most clinical cases, there is no need for surgery on soft tissues, which eliminates the possibility of additional traumas.

**Conclusions:**

The use of a promising technology for the fabrication of a crown on the implant and a customized provisional composite abutment significantly reduced the period of orthopedic rehabilitation of the patient. Immediate implantation with a customized provisional composite abutment completely forms the gingival profile, reduces the risk of microbial contamination in the area of bone formation, minimizes soft tissue ischemia, and accelerates the processes of mucogingival and bone integration around the implant.

## 1. Introduction

The development of implantology urges the specialists to reduce the time of implant integration and prosthetic procedures and to quickly and successfully restore the patient [1–6].

At present, there is a sharp increase in interest in immediate or early loading of the implant, when certain parameters of the primary implant stability in the bone tissue are achieved [7–11].

With the initial implant stability of 35 N/cm^2^ and higher, it is possible to immediately load the implant with a provisional prosthesis, which makes it possible to quickly restore the patient within a few hours after surgery [12–17].

Achieving the primary implant stability of 25–30 N/cm^2^ does not always make it possible to install the provisional prosthesis; its hasty installation can lead to a violation of the bone-implant integration and subsequently to implant loss [18–21].

**Figure 1:**
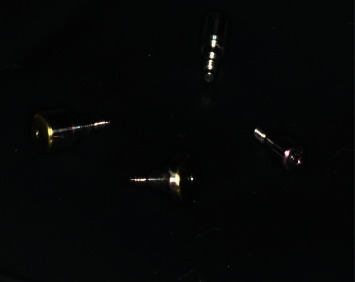
Gingiva formers of various diameters.

Therefore, surgeons often leave the implant without loading, commonly applying standardized round gingiva formers of various lengths and diameters, used on the day of surgery and located in the implant until the prosthesis is fixed. As a rule, the formers do not meet the requirements of the future prosthesis and do not replicate its shape and anatomy (Figure 1).

These drawbacks can be eliminated by using a customized provisional prosthesis tightly fitting the soft tissues, which could replicate the anatomy of the future permanent prosthesis.

One of the methods for fabricating a customized gingiva former is used for immediate implantation in grinder teeth [22].

After the tooth extraction and implant placement in the correct orthopedic position, a bone xenograft is placed between the cortical plate of the extraction socket and the implant. Then, intraoral scanning and modeling of a customized gingiva former in the program InLab Sirona “Laboratoire Eric Berger” focusing on the soft tissues and adjacent teeth are performed. During fabrication, the patient is fitted with a standardized gingiva former.

The foundation for the fabrication of a customized former was a Ti-Base for permanent zirconia prostheses and PEEK (BREDENT) material glued onto the adhesive cement. After the former was fabricated, it was glued into the titanium base and placed into the implant with a torque force of up to 15 N/cm^2^ under the control of an X-ray image.

The time for modeling and fabrication of a customized former was several hours. After the final implant integration, the customized former was removed and the permanent prosthesis was fabricated using the digital method.

The following are the drawbacks of this technology:

- High cost of materials
- Large time costs connected with the modeling and fabrication of a customized former in a digital laboratory and the subsequent gluing of the PEEK material into a titanium base
- Prolonging of the surgical stage due to the patient's waiting in the operating room and the fabrication of a customized former in the dental laboratory
- Additional time for sterilizing the product after fabrication

Therefore, the development of a technology for the fabrication of a customized provisional composite abutment, which is installed intraoperatively, is relevant.

The aim of the work is to quickly form the required emergence profile of the future prosthesis using the developed customized provisional composite abutment screwed to the implant immediately after its placement.

## 2. Materials and Methods

We studied 20 patients, divided into an experimental group consisting of 9 people (implantation with the use of a customized provisional composite abutment)—of which 5 patients had a delayed implantation with implant placement into mature bone and 4 patients underwent immediate implantation after tooth extraction—and an comparison group consisting of 11 people (implantation with the use of a prefabricated gingiva former)—of which 10 patients underwent delayed implantation in mature bone and one had a one-stage implantation immediately after tooth extraction. The quantitative characteristics of the patients are presented in Table 1.

Patients were included in the study according to the criteria presented in Table 2.

### 2.1. For the Developed Technology

**Figure 2:**
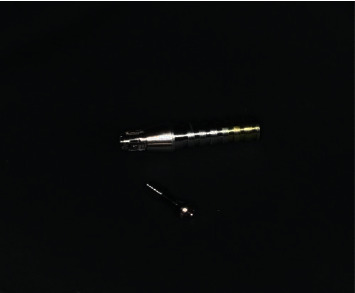
Prefabricated provisional abutment.

A customized provisional composite abutment is fabricated directly before the dental implantation surgery, at the planning stage. The basis for the item is a provisional prefabricated abutment, the neck of which varies from one to three millimeters and smoothly turns into a narrowing—a shoulder and a retention grip for the composite (Figure 2).

For better adhesion of the composite, the retention grip of the provisional abutment is coated with 27 *μ*m aluminum oxide powder using the apparatus RONDOFLEX (KERR). Gluing of the composite to the surface of the provisional abutment is carried out using the Single Bond Universal 3M light adhesive by applying it to the provisional abutment and light polymerization.

The composite (Filtek Bulk Fill 3M) contains a low-viscosity X-ray contrast nanocomponent with a filler (ytterbium trifluoride) with a particle size of 0.01–3.5 nm. It has excellent polishing properties and good wear resistance in comparison with other composites; it makes it possible to polymerize the material with a thickness of more than 4 mm and has low shrinkage. Uniform polymerization and hardening of the material are carried out with a Demi Ultra Kerr lamp with a luminous power of at least 1100 mW/cm^2^.

**Figure 3:**
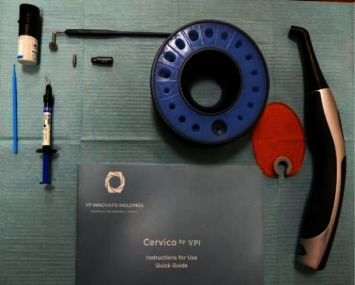
Cervico system.

The customized provisional composite abutment is fabricated using the Cervico system. The upper ring of the device is rotated until the desired size of the depression matches the required size of the desired prosthetic connection at the base of the device. This information is recorded in a special form, which in the future may be necessary for the orthopedic management of the patient. In addition to the selection of the shape for the future emergence profile, the Cervico device allows one to set the depth of implant immersion in the bone of 0–4 mm (Figure 3).

**Figure 4:**
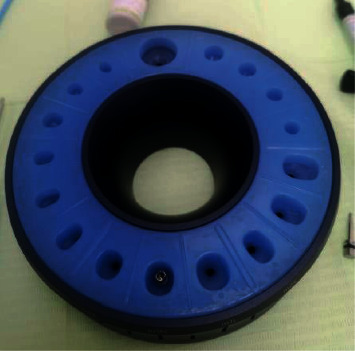
Placement of the prefabricated abutment into the Cervico device.

After the final setting in the Cervico system of the necessary parameters for the fabrication of a customized provisional abutment, an analogue of the corresponding implantation system is fixed in the device. Its diameter is completely identical to the dental implant, which will be inserted into the bone tissue. The provisional abutment in the analogue is fixed with an occlusal screw with a torque force of up to 15 N/cm^2^ (Figure 4).

**Figure 5:**
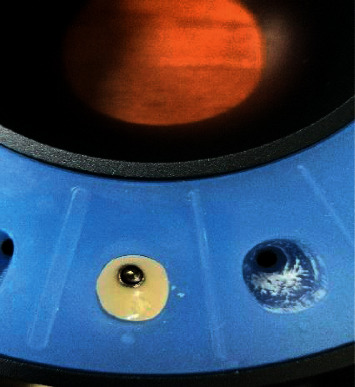
Stage of introducing the composite and polymerization.

A fluid light composite is introduced into the selected cell and illuminated with a polymerization lamp (Figure 5).

**Figure 6:**
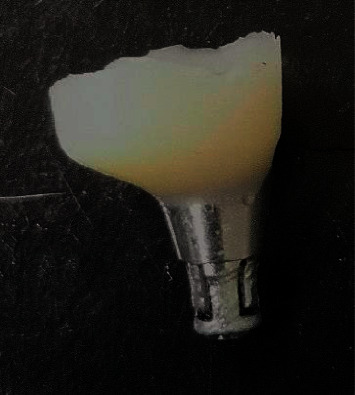
Customized provisional composite abutment.

After the composite has hardened, the customized provisional composite abutment is removed from the Cervico device; the emergence profile is additionally finished to ensure a smooth transition from the abutment neck to the composite and then polished (Figure 6). After preliminary assessment of the occlusal position, it is necessary to shorten the customized provisional composite abutment to the antagonist teeth.

A customized provisional composite abutment can be fabricated in advance before the surgery and then sterilized in an autoclave at a pressure of 1.1 atm and a temperature of 120°C for 45 minutes.

### 2.2. For Conventional Technology

The standardized gingiva former is a titanium cylinder with a screw for insertion into the implant and is used for the formation of soft tissues before the prosthetic stage. The gingiva former of many implant systems is available in diameters from 3 to 9 mm (incisal, premolar, molar) and lengths from 1 to 7 mm. Most are conical and cylindrical in shape. They are used for placement in an implant immediately, in case of achieving good primary implant stability, or after some time, with delayed implantation. In the latter case, an incision is made in the mucous membrane, an implant is found, the plug of the implant is unscrewed, the implant shaft is washed with an irrigation solution, and a gingiva former is placed, which is selected depending on the thickness of the mucosa that should rise no more than 1–2 mm above the gingiva. The gingiva former is necessary for the formation of soft tissues around the implant and for quick access to it during the prosthetics stage. The torque force when it is inserted into the implant is set to no more than 10 N/cm^2^.

The standardized gingiva former does not completely recreate the contours of the future prosthesis and requires additional shaping of the gingiva with a provisional crown fabricated in a dental laboratory.

In addition, there are laboratory methods for the fabrication of customized titanium formers by the analogue method, as well as current methods using CAD/CAM digital technologies.

### 2.3. Statistical Analysis

The end point of the study was to determine the time of prosthetics from the digital impression of the gingival profile to the final orthopedic rehabilitation for the developed technology and the conventional approach using a prefabricated gingiva former. The implant was used as a statistical unit and analyzed. Statistical analysis was performed using NCSS 2020 software. Standard descriptive methods such as median, frequency, minimum, and maximum were used to determine sample characteristics. Quantitative data were compared between the groups using the Mann–Whitney *U* test and within groups using the Wilcoxon test to assess the normality of the distribution. The confidence interval was set at 95%.

## 3. Results

All 20 patients underwent dental implant surgery with primary stability of 25 to 45 N/cm^2^.

Eleven patients received prefabricated gingiva formers, ten of which had implants integrated into the mature bone. One patient received a prefabricated gingival former inserted into the implant immediately after tooth extraction.

Nine patients had a customized provisional composite abutment placed in the implant:

- Immediately after tooth extraction—4 patients
- Delayed, in the mature bone—5 patients

**Figure 7:**
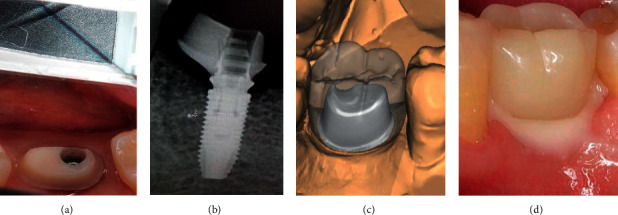
Stages of application of a customized composite abutment with immediate loading: (a) preparation, (b) checking the fit, (c) modeling of the provisional crown in the program, and (d) placement of the provisional crown.

The technique of fabrication and placement of the provisional composite abutment for one-stage implantation immediately after tooth extraction included the following stages (Figure 7):

- After the placement of a customized provisional composite abutment into the implant area intraoperatively, within 72 hours, its supragingival preparation using a turbine tip is performed
- Scanning with a 3Shape intraoral scanner of a provisional composite abutment
- Modeling of the framework of a provisional prosthesis and subsequent fabrication of a provisional CAD/CAM crown from a PMMA (polymethyl methacrylate) block
- Treatment of a customized provisional composite abutment with adhesive
- Introducing a light composite into a provisional crown and gluing onto a customized composite abutment
- Final polymerization (occurs within 20 seconds)

**Figure 8:**
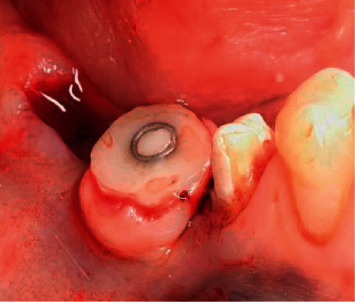
Intraoperative placement of a provisional composite abutment.

After the placement of the dental implant into the bone in the correct orthopedic position, including with respect to the plane of the future suprastructure, a customized provisional composite abutment is placed on the implant in the oral cavity with a torque force on the screw of up to 15 N/cm^2^ (Figure 8).

**Figure 9:**
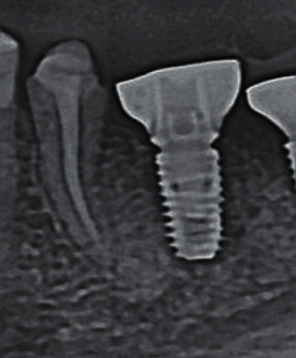
Checking the fit of the provisional composite abutment.

The fit of the provisional abutment to the implant is checked using an X-ray image (Figure 9). The shaft opening of the provisional abutment is closed with a Teflon tape and sealed with a light composite. After the suprastructure has been placed, nonresorbable sutures are placed to hold the flap around the customized provisional composite abutment.

In the absence of primary implant stability (less than 25 N/cm^2^), the placement of a customized provisional composite abutment is performed 2–6 months after the final implant integration is achieved.

After the customized provisional composite abutment has formed the emergence profile and the implant has achieved the required final integration, as a rule, with early loading, there follows the transition to the stage of fabrication of a permanent prosthesis, bypassing the provisional crown, using digital technologies.

**Figure 10:**
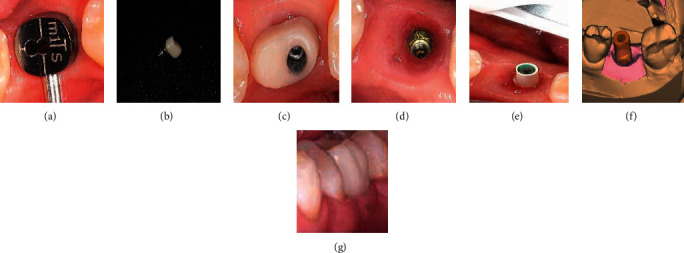
Stages of fabrication of a permanent prosthesis after the formation of the emergence profile with a customized composite abutment: (a) planning, (b) a customized composite abutment, (c) the abutment placement (3 weeks after surgery), (d) formation of the emergence profile (10 weeks), (e) intraoral scanning, (f) planning of a permanent prosthesis in the program, and (g) placement of a permanent prosthesis.

Once the soft tissues have been formed, the provisional composite abutment is carefully removed with a torque key and the emergence profile is assessed. A scan marker is installed in the implant to scan the area of the formed emergence profile. The special program simulates a permanent implant-supported prosthesis. The prosthesis is fabricated of a biocompatible material—zirconium dioxide within three hours. A customized provisional composite abutment is removed from the implant, the antiseptic irrigation of the internal shaft of the implant is performed, and the permanent prosthesis is placed with a torque force of at least 30 N/cm^2^ (Figure 10).

All this makes it possible in a few hours to provisionally restore a patient after surgery.

**Figure 11:**
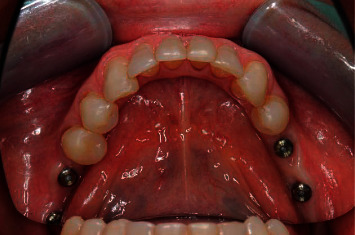
Placement of gingiva formers.

**Figure 12:**
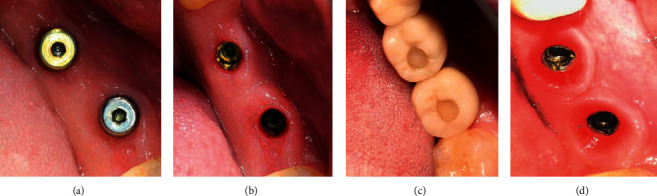
Stages of soft tissue formation: (a) immediately after surgery, (b) after 6 to 8 weeks (with prefabricated gingiva former), (c) after 10 weeks (with provisional crowns), and (d) after 12 weeks of provisional crowns.

The prefabricated gingiva former used in classical delayed implantation in mature bone (Figure 11) has a small diameter, which leads to the need for additional soft tissue formation with a provisional crown within 7–14 days (Figure 12).

The time of placement of the dental prosthesis in the implant ranged from 24 hours to 3 months. After 6–8 weeks, the X-ray control of the implant integration and assessment of the emergence profile were performed.

## 4. Discussion

The use of a standard gingiva former for immediate implantation leads to the need to suture the soft tissues with tension around the former, immobilizes the flap with additional trauma, and, subsequently, results in soft tissue deficiency.

**Figure 13:**
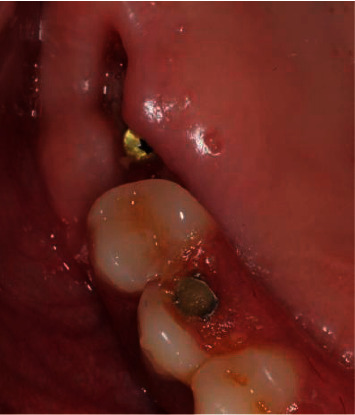
Microbial contamination of soft tissues.

In particular, in immediate implantation in the masticatory system, the space that is formed between the former and the gingival flap negatively affects the tightness and, subsequently, can cause delayed microbial contamination around the implant, which is what happened to one patient (Figure 13).

Lack of proper sealing can lead to loss of a clot or bone material placed in the space between the cortical plate and the implant. Tissue healing often occurs by secondary intention.

In both groups of patients, X-ray images were made directly during the surgery to check the accuracy of the placement of the superstructure with respect to the implant platform.

All participants were prosthetized with a provisional and, subsequently, with a permanent prosthesis, depending on the primary bone stability of the implant.

Of the nine patients in the experimental group, 5 patients received early loading in the form of a provisional prosthesis after the healing stage and final integration of the implant (6–8 weeks). The remaining four patients received early (2 patients) and immediate (2 patients) loading with a provisional crown on the day of surgery.

**Figure 14:**
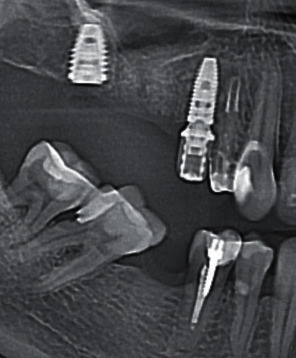
Bone regeneration after 6 weeks.

At the stage of examination of the patients who underwent classical delayed implantation with a customized provisional composite abutment (5 patients), on the 3rd–5th day, it was determined that the wound healing occurred by primary intension and the sutures were good. They were removed on the 10–14th day. The epithelization of soft tissues was complete, and no inflammation was detected. X-ray images taken to check the integration after 6 weeks showed no abnormalities in bone regeneration around the implants (Figure 14).

It is worth noting the absence of soft tissue inflammation around the implants in four patients from the experimental group; the implants were placed together with a customized provisional composite abutment immediately after tooth extraction.

**Figure 15:**
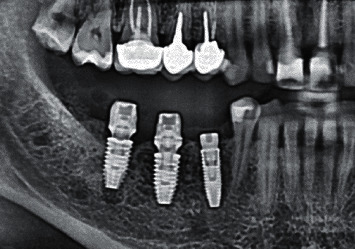
Formation of the bone matrix.

X-ray images show the formation of the bone matrix after 6 weeks (Figure 15).

Objectively, the soft tissues around the implant placed immediately are stable, tightly adhering to the customized provisional composite abutment. The sutures in 5–7 days after the implant placement are good; no tissue inflammation was detected.

In the comparison group, consisting of 11 patients, a dental implantation surgery was performed and prefabricated gingiva formers were placed. Ten of them underwent delayed implantation in mature bone. One person in this group had an implant placed immediately after a tooth extraction. The primary stability of the implants ranged from 25 to 30 N/cm^2^.

**Figure 16:**
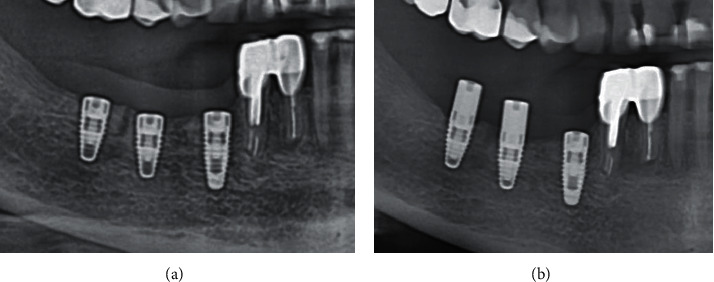
Dynamics of bone resorption after the placement of a gingiva former: (a) the moment of surgery and (b) 6 weeks.

X-rays after 6 weeks showed marginal resorption in one of 10 patients with an implant in the mature bone with a gingiva former (Figure 16).

In 11 patients, the implants were placed in mature bone and the delayed implantation with early loading after 6–8 weeks was carried out. To shape the emergence profile, a provisional milled crown was fabricated in the dental laboratory.

The remaining four patients underwent immediate postextraction implantation with the placement of prefabricated gingiva formers. Immediate loading with the prosthesis on the implants on the day of surgery was not performed.

The only patient in the comparison group who underwent immediate implantation with a gingiva former showed the following features on day 4–5 of the postoperative examination:

- Soft tissues around the implant do not fit tightly in the zone of the gingiva former
- The wound surface healing occurs by secondary intention

Comparative characteristics of positive and negative aspects of the conventional and new methods are shown in Table 3.

The risk of bacterial contamination of the implant area after the placement of prefabricated gingiva formers is higher than that when using a customized provisional composite abutment. It is not always possible to achieve guaranteed success in soft tissue healing around gingiva formers.

The use of gingiva formers at the prosthetic stage is accompanied by the formation of a small-diameter gingival profile. Subsequently, after the final implant integration, this leads to additional formation of the gingival profile by a provisional crown, which increases the time of prosthetics.

The main minor disadvantage of using a customized provisional composite abutment is the increased adhesion to the plaque due to the greater specific surface area of the porous, slightly roughened structure.

This disadvantage is more than compensated by the advantages of a customized provisional abutment:

- The ability to seal the soft tissue-bone space
- No additional immobilization of tissues at the stage of suturing a tooth socket in the case of immediate implantation
- Quick formation of the necessary gingival profile, taking into account the shape of the future prosthesis
- No need for a provisional laboratory crown
- Quick orthopedic rehabilitation of the patient

Table 4 shows comparative data on the timing of the final soft tissue formation for prosthetics in patients in the experimental group and the comparison group.

The above data show that the developed technology makes it possible to spend much less time for orthopedic rehabilitation of patients. In the experimental group, there was a statistically significant decrease in the time from delayed implantation to one-stage implantation (Wilcoxon sign-rank test; *p* < 0.01). However, there was no significant difference between timing depending on implant placement—mandible or maxilla—in either group (Wilcoxon sign-rank test, *p* > 0.05; Mann–Whitney *U* test; *p* > 0.05).

In the comparison group, the timing did not virtually depend on the type of implantation—delayed or single—stage (Wilcoxon sign-rank test, *p* > 0.05) and was at least 360 hours. This postpones the moment of orthopedic rehabilitation of the patient, which can be avoided when using a customized provisional composite abutment fabricated with the Cervico system.

## 5. Conclusions

The use of modern technologies for crown fabrication on an implant by a direct digital method and the application of a customized provisional composite abutment made it possible to significantly reduce the time of the patient's prosthetic rehabilitation. Other advantages of the developed technology are the reduction in bacterial contamination in the bone formation zone, minimization of soft tissue ischemia, acceleration of mucogingival and bone integration, and rapid formation of the desired emergence profile of the future prosthesis.

## Figures and Tables

**Table 1:** Quantitative characteristics of the patients according to age, gender, and nosology.

Diagnosis	Primary implant stability (N/cm^2^)	Age (years)	Dental implants		
			Gender		Total amount
			М (tooth number)	F (tooth number)	
Partial edentulous maxilla delayed implantation (early loading with a prosthesis)	25–35	38–54	3 (26, 17, 17)	5 (25, 26, 16, 16, 17)	8

Partial edentulous mandible delayed implantation (early loading with a prosthesis)	25–35	42–56	2 (36, 46)	5 (44, 47, 36, 36, 37)	7

Partial edentulous maxilla delayed implantation (immediate loading with a prosthesis)	35–45	41–46	1 (24)	1 (25)	2

Partial edentulous maxilla (immediate postextraction implantation—early loading with a prosthesis)	30–35	34–40	1 (14)	—	1

Partial edentulous maxilla (immediate postextraction implantation—early loading with a prosthesis)	30–35	38–45	—	2 (46, 47)	2

**Table 2:** Inclusion and exclusion criteria for the participants.

	Inclusion criteria	Exclusion criteria
General	(i) Age > 21 years (ii) Absence of medical comorbidities (iii) Absence of periodontal diseases (iv) Antagonist dentition (v) Availability for 20-week follow-up	(i) Inadequate oral hygiene (ii) Smoking
Local	(i) Missing 1 or 2 teeth (included oral distal defects of the masticatory system—molars and premolars) (ii) In the case of one-stage implantation, teeth with more than 80% decay or 3–4° mobility (iii) Plaque indicators throughout the oral cavity and bleeding indicators of less than 25%	(i) Adjacent teeth with the presence of carious processes (ii) Presence of periapical inflammation on adjacent teeth (iii) Local inflammation of the periodontium (iv) Mobility of adjacent teeth (v) Mucosal disease

**Table 3:** Comparative characteristics of positive and negative aspects of the conventional and new methods.

Parameters	Comparison group with gingiva former (11 patients)		Experimental group with provisional composite abutment (9 patients)	
	Placement in mature bone (10 patients)	Immediate placement in implant after tooth extraction (1 patient)	Placement in mature bone (5 patients)	Immediate placement in implant after tooth extraction (4 patients)
Microbial contamination	+ (1 patient)	+	–	–
Bone augmentation loss	–	+	–	–
Narrow gingival profile requiring further shaping	+	+	–	–
Increased adhesion to plaque	–	–	+	+
The need for additional tissue immobilization	–	+	–	–
Tight sealing of the soft tissue-bone space	+	–	+	+
Quick formation of the necessary gingival profile (according to the shape of the tooth)	–	–	+	+
Increased requirements for the dentist's manual skills	–	–	+	+
The possibility of obtaining additional tissue volume through grafting	–	–	+	+
Saving orthopedic rehabilitation time (7–21 days)	–	–	+	+

*Note.* +: yes; −: no.

**Table 4:** Timing of the final orthopedic rehabilitation.

Patients		Number of implants and time of prosthetics in hours (from digital impression of the gingival profile to final orthopedic rehabilitation)			
		Delayed implantation (early loading)		One-stage implantation (immediate and early loading)	
		M	F	M	F
Experimental group	Mandible	1–84 ± 12	1–120 ± 12	—	2–72 ± 8
	Maxilla	1–104 ± 18	2–96 ± 12	1–60 ± 8	1–84 ± 10
Comparison group	Mandible	1–432 ± 72	4–360 ± 60	—	—
	Maxilla	2–384 ± 68	3–360 ± 60	1–408 ± 72	—

## Data Availability

The data used to support the findings of this study are available within the article.
